# Luteolin: exploring its therapeutic potential and molecular mechanisms in pulmonary diseases

**DOI:** 10.3389/fphar.2025.1535555

**Published:** 2025-02-12

**Authors:** Jialian Lv, Xinyue Song, Zixin Luo, Duoqin Huang, Li Xiao, Kang Zou

**Affiliations:** ^1^ The First Clinical Medical College, Gannan Medical University, Ganzhou, Jiangxi, China; ^2^ Department of Rehabilitation Medicine, The First Affiliated Hospital of Gannan Medical University, Ganzhou, Jiangxi, China; ^3^ Department of Critical Care Medicine, The First Affiliated Hospital of Gannan Medical University, Ganzhou, Jiangxi, China

**Keywords:** luteolin, pulmonary diseases, molecular mechanisms, bioavailability, pharmacokinetics, pharmacological activities

## Abstract

Luteolin is a flavonoid widely found in plants, including vegetables, botanical drugs, and fruits. Owing to its diverse pharmacological activities, such as anticancer, oxidative stress protection, anti-inflammatory, and neuron-preserving effects, luteolin has attracted attention in research and medicine. Luteolin exhibits therapeutic effects on various pulmonary disease models through multiple molecular mechanisms; these include inhibition of activation of the PI3K/Akt-mediated Nuclear Factor kappa-B (NF-κB) and MAPK signaling pathways, as well as the promotion of regulatory T cell (Treg) function and enhancement of alveolar epithelial sodium channel (ENaC) activity (alleviating inflammation and oxidative stress responses). Luteolin has therapeutic effects on chronic obstructive pulmonary disease (COPD), acute lung injury/acute respiratory distress syndrome (ALI/ARDS), pulmonary fibrosis, allergic asthma, and lung cancer. Luteolin, a naturally occurring polyphenol, is poorly water-soluble. The oral route may be ineffective because the gut poorly absorbs this type of flavonoid. Therefore, although luteolin exhibits significant biological activity, its clinical application is limited by challenges associated with its poor water solubility and low bioavailability, which are critical factors for its efficacy and pharmacological application. These challenges can be addressed by modifying the chemical structure and enhancing pharmaceutical formulations. We summarized the research advancements in improving the solubility and bioavailability of luteolin, as well as the effects of luteolin on various pulmonary diseases and their related mechanisms, with the aim of providing new ideas for researchers.

## 1 Introduction

Pulmonary diseases are one of the major health issues worldwide, with high incidence and mortality rates (Chen and Li, 2024; Meng et al., 2025; GBDCRD, 2023). According to the World Health Organization, COPD, asthma, pulmonary fibrosis, lung cancer, and other pulmonary diseases lead to millions of deaths each year (Roh, 2024; Wang et al., 2025; Podolanczuk and Raghu, 2024; Lu et al., 2025). With the aging of the population and the intensification of environmental issues, the burden of pulmonary diseases is expected to increase further (Faniyi et al., 2022). Although there are various drugs available for the treatment of pulmonary diseases, there are still many challenges, such as drug side effects, drug resistance, and the lack of effective treatments for certain diseases (Mazumder et al., 2024; Dorababu and Maraswami, 2023; Bandyopadhyay and Mirsaeidi, 2023).

Against this backdrop, the potential of natural products in the treatment of pulmonary diseases is increasingly gaining attention. Natural products, with their rich chemical diversity and biological activity, offer valuable resources for drug development. Compared with traditional chemical drugs, natural products often have better biocompatibility and lower toxicity, and have shown unique advantages in regulating the body’s immune system, anti-inflammatory, and antioxidant aspects (Li C. et al., 2024; Wang et al., 2023). From traditional Chinese medicine to modern botanical drugs, the application of natural products in the treatment of pulmonary diseases has a long history, and new research results are constantly emerging (Rahman et al., 2022).

Currently, there are numerous narrative reports about luteolin in various diseases (Zhang et al., 2024; Shi et al., 2024; Huang L. et al., 2023; Diniz et al., 2022), but most of the research on pulmonary diseases focuses on describing its therapeutic effects, with few exploring and summarizing its potential mechanisms. This study summarizes the effects of luteolin on various pulmonary diseases and related mechanisms, aiming to provide new insights for researchers and to promote further research and application of luteolin in the field of pulmonary disease treatment.

## 2 Plant sources of luteolin and its physicochemical properties

**FIGURE 1:**
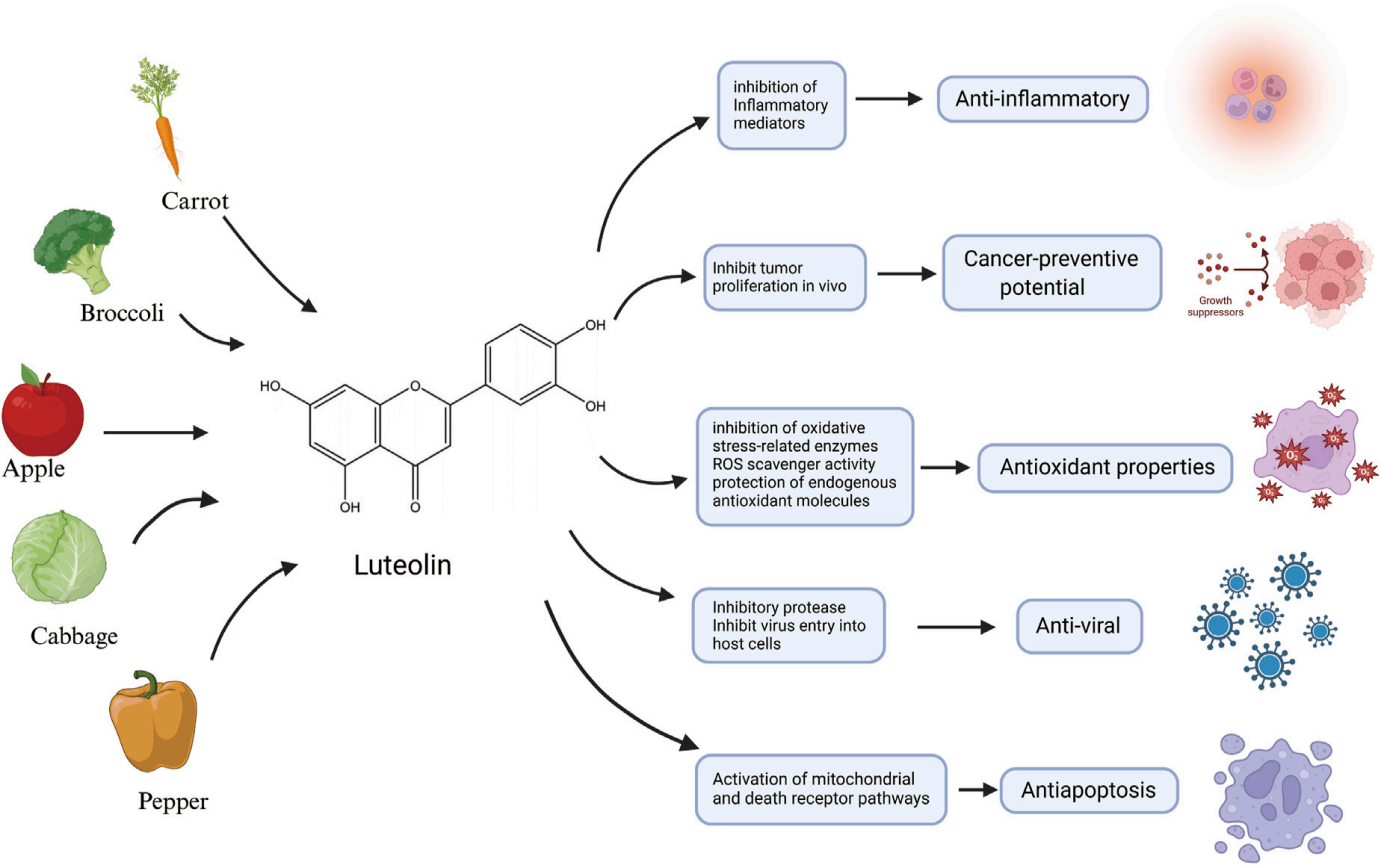
An illustration of the main source and anti-inflammatory, anticancer potential, antioxidant properties, antiviral activity and antiapoptotic characteristics of luteolin, created using Biorender.com.

Over the last 2 decades, there has been mounting emphasis on utilizing metabolites derived from medicinal plants to treat and manage a diverse array of diseases (Xiong et al., 2021; Ahmad et al., 2020; Singh et al., 2023; Ramadaini et al., 2024). Luteolin, an active flavonoid metabolite isolated from *Lonicera japonica*, exhibits many biological activities. It is also known for its potent antioxidant and anti-inflammatory characteristics (Xiong et al., 2024; Jia et al., 2023). Plants abundant in luteolin have been used in traditional Chinese medicine for treating a wide array of illnesses, including hypertension (Gao et al., 2023), various inflammatory conditions (Attiq et al., 2021), and cancer (Jiang et al., 2021). In addition to its medicinal use, luteolin is also present in various vegetables, such as fresh *Spinacia oleracea*, *Petroselinum crispum*, and *Brassica oleracea* var. *Capitata,* and a range of fruits and botanical drugs (Manzoor et al., 2019; Almatroodi et al., 2024). Flavonoids—prevalent polyphenolic metabolites within the plant kingdom—have demonstrated health benefits in humans at particular concentrations (Wen et al., 2021). These metabolites are biosynthesized via the phenylpropanoid pathway in plants (Cocuron et al., 2019). Luteolin has a classic flavonoid structure featuring four hydroxyl groups at 5′, 7′, 3′, and 4′positions. The structural hallmark of luteolin includes two aromatic rings, A and B, which are linked to an oxygenated pyran ring labeled as C. The biological activities of luteolin are potentially contingent upon the presence of its hydroxyl groups and the double bond that exists between carbons 2 and 3. Typically, luteolin is encountered in a glycosylated form, where one or more of its hydroxyl groups have been replaced by diverse glycosyl groups (Huang L. et al., 2023). Similar to other flavonoids, luteolin is an antioxidant and can prevent damage to DNA, lipids, and proteins induced by reactive oxygen species (ROS) (Zou et al., 2021). Molecules such as hydrogen peroxide (H_2_O_2_), singlet oxygen, and hydroxyl radicals are highly reactive with numerous biological targets and play crucial roles in neuromodulation, immunomodulation, ion transport, and apoptosis (Mittler, 2017). Luteolin influences the activities of various enzymes associated with oxidative stress. Among these, oxidases responsible for the generation of ROS, such as xanthine oxidase, are inhibited by luteolin (Liu X. et al., 2022). Luteolin also inhibits the activity of enzymes that catalyze the oxidation of cellular constituents, including cyclooxygenases and lipoxygenases (Han et al., 2024; Ren et al., 2021). Additionally, luteolin can be oxidized and serves as a scavenger of ROS—an activity that stems from the chemical structure it shares with other flavonoids, enabling it to provide hydrogen atoms from the hydroxyl groups present on its aromatic rings (Ramesh et al., 2021). Despite the promising outlook on the therapeutic characteristics of luteolin, it is imperative to consider its possible toxic effects and broader health implications (Galati and O'Brien, 2004; Khan et al., 2021). Understanding the full spectrum of its effects on human biology is crucial for its effective use, as shown in Figure 1.

### 2.1 Pharmacokinetics of luteolin

#### 2.1.1 Bioavailability

The *in vivo* bioavailability of luteolin is relatively low owing to its poor stability and low absorption rate, which consequently diminishes its therapeutic effectiveness. The bioavailability of total luteolin, including free and bound forms, has been reported to be 53.9%. Despite an apparent good absorption rate, the level of free luteolin present in the systemic circulation is lower than that of its metabolites, with the actual bioavailability of free luteolin being merely 17.5%. This suggests that luteolin is promptly absorbed upon entering the rat intestinal system and undergoes comprehensive metabolism in the intestinal and/or hepatic cellular structures, which is the reason for its low bioavailability (Yao et al., 2023; Deng et al., 2017). Following oral administration, luteolin achieves its peak plasma concentration within 1–2 h and is retained in circulation for several hours thereafter. Its metabolism is sufficiently slow to allow the metabolite to exhibit biological activity *in vivo* (Ali and Siddique, 2019). When comparing the absorption efficiency of a single oral dose of pure luteolin or luteolin derived from peanut hull extract, the jejunum and duodenum had higher absorption rates than the colon and ileum. Furthermore, the bioavailability of luteolin from the peanut hull extracts significantly surpassed that of pure luteolin (Zhou et al., 2008). Upon absorption, luteolin is rapidly taken up, and can be detected in the plasma as luteolin, luteolin monoglucoside, and luteolin glucuronide, along with sulfate conjugates of luteolin aldehyde and O-methylated luteolin (Yasuda et al., 2015; Shimoi et al., 1998). Certain co-occurring metabolites, such as eriodictyol, enhance the absorption of luteolin in the intestine, thereby improving its bioavailability (Zhou et al., 2008). Moreover, various pharmaceutical techniques, including cyclodextrin complexation, luteolin-loaded nanocarriers, polymeric micelles, phospholipid complexation, RA (rosmarinic acid)-SS-mPEG, and combined hydroxyethyl starch luteolin nanocrystals have been used to augment the solubility and bioavailability of luteolin. These methods slow the degradation of luteolin in the bloodstream, thereby prolonging its circulation time (Liu et al., 2013; Khan et al., 2016; Qing et al., 2017; Kar et al., 2024; Luo et al., 2024; Lu et al., 2023; Pittol et al., 2022). Recently, Imam and colleagues prepared luteolin-loaded nanovesicles (NVs) using the solvent evaporation method, and antioxidant activity results demonstrated that luteolin-NVs exhibited greater activity than pure luteolin. Cytotoxicity studies have shown that the IC50 value of luteolin NVs was lower than that of pure luteolin (Imam et al., 2022). Miao et al. formulated luteolin-loaded methoxy poly (ethylene glycol)-polylactide micelles (luteolin/MPEG-PLA) to enhance the bioavailability of luteolin in pulmonary infectious diseases (Miao et al., 2021). Ren et al. improved the bioavailability of luteolin four-fold using a cyclodextrin MOF-modified dry powder inhaler to treat fibrotic interstitial lung disease (Ren et al., 2023). These studies illustrate that strategies for improving the solubility and bioavailability of luteolin through structural modifications and formulation advancements are likely to facilitate its use in the treatment of diseases in the future.

#### 2.1.2 Distribution

**FIGURE 2:**
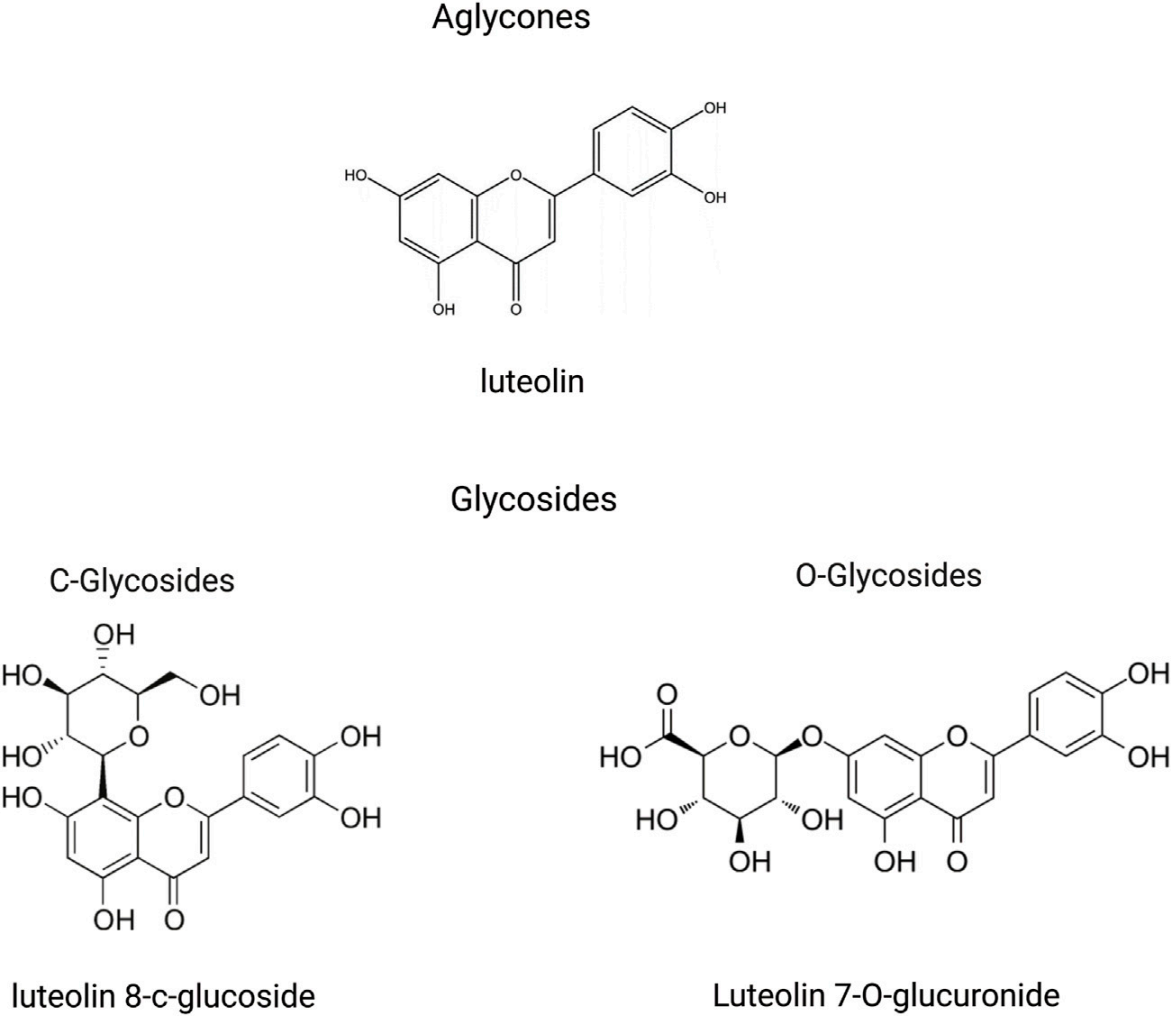
An illustration of structural formulas (through the free hydroxyl groups to form O-glycosides and through the formation of a carbon-carbon bond to create C-glycosides) of metabolites related to luteolin, created using Biorender.com.

In the plant kingdom, luteolin is commonly found in a glycosylated state. During absorption, these glycosides are hydrolyzed to yield the free form of luteolin (Wang Z. et al., 2021). Glycosylation of the aglycone moiety primarily occurs via two distinct mechanisms: free hydroxyl groups to form O-glycosides and forming a carbon-carbon bond to create C-glycosides (Figure 2) (Hostetler et al., 2017; Punia Bangar et al., 2023). Following absorption, luteolin and its metabolites are rapidly and widely distributed throughout the major organs and tissues of the body. Throughout the rat circulatory system, luteolin is largely present in conjugated forms, with luteolin-glucuronide being the most prevalent metabolite circulating in most tissues (Hayasaka et al., 2018). In the current study, luteolin was highly concentrated in the liver, kidneys, gastrointestinal tract, and lungs in conjunction with its multiple metabolites. Importantly, luteolin can cross the blood-brain barrier, suggesting its potential for treating central nervous system diseases (Daily et al., 2021). These findings support the action of luteolin on these medicinal targets (Chen et al., 2010; Domitrović et al., 2009; Lu et al., 2015; Xu et al., 2015). Because of their ability to cross the placenta, these substances may be toxic to the fetus in the womb (Skibola and Smith, 2000).

#### 2.1.3 Elimination

**FIGURE 3:**
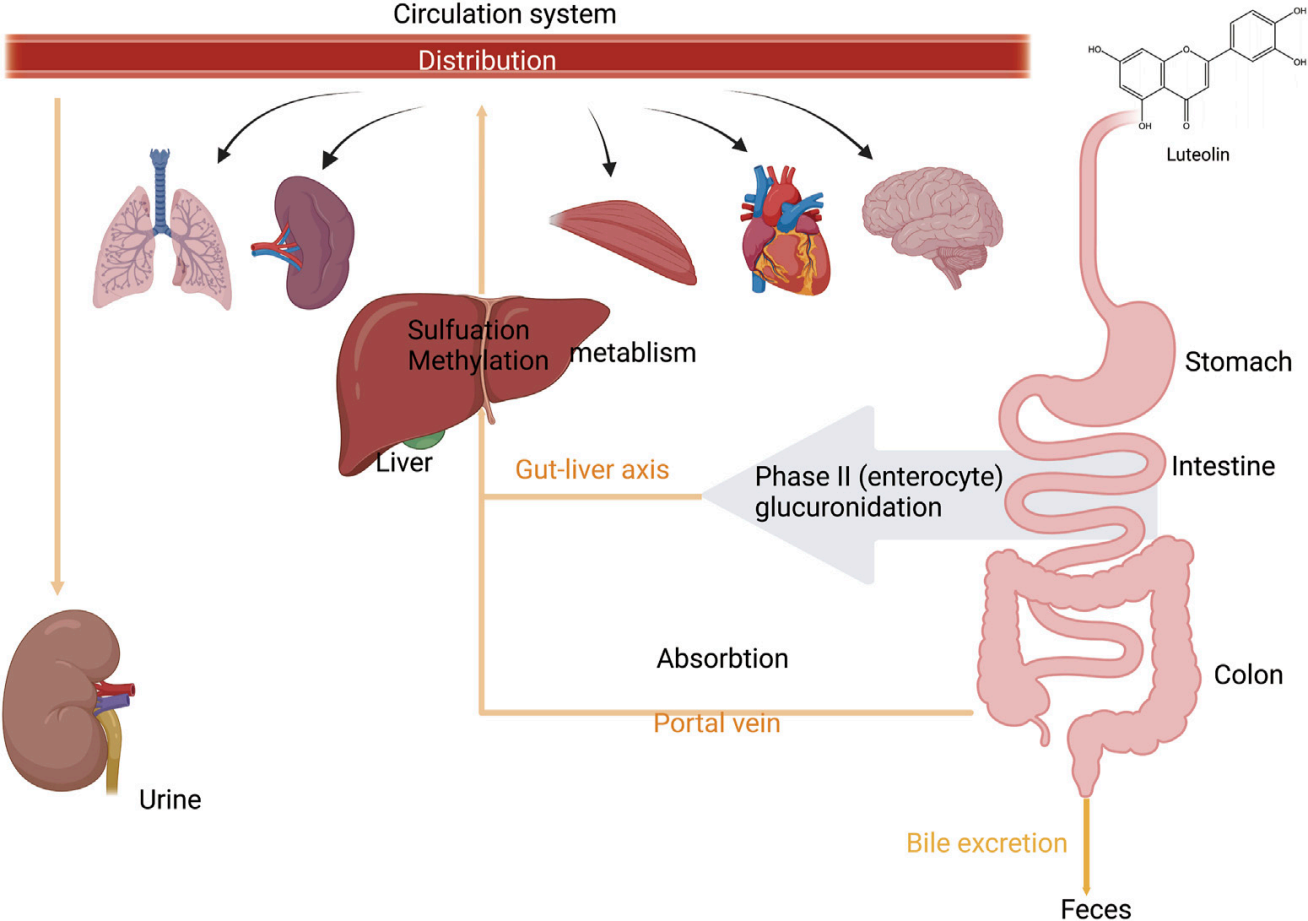
An illustration of absorption, distribution, and metabolism of luteolin. Luteolin is absorbed from the intestine and enters the liver via the portal vein. During the first phase of metabolism, it is metabolized in the intestinal wall and/or by liver cells. In the second phase of metabolism, it undergoes enterohepatic recirculation. It is then distributed to various organs and tissues throughout the body via the bloodstream and is finally excreted through urine and bile, created using Biorender.com.

After absorption, flavonoids undergo metabolic processes in the liver, including glucuronidation, sulfation, and methylation. In addition, they can be metabolized into smaller phenolic metabolites (Bravo, 1998). Specifically, luteolin is first metabolized through glucuronidation or methylation and converted into a methylated glucuronide form. The principal Phase II metabolite has been confirmed to be luteolin-3′-O-glucuronide (Boersma et al., 2002). Luteolin-3′-O-glucuronide is the predominant metabolite in these metabolic pathways (Kure et al., 2016). Furthermore, two novel diglucuronide conjugates of luteolin were separated by incubation with uridine diphosphate glucuronosyltransferase (UGTs). Methylation has also been identified as a significant metabolic pathway for luteolin. This methylation process produces chrysoeriol and diosmetin, a reaction facilitated by catechol-O-methyltransferase in rats (Wu et al., 2015; Chen et al., 2011; Wang et al., 2017). Upon oral ingestion, luteolin is quickly absorbed into the systemic circulation and undergoes extensive metabolic processing within the rat body. The recovered metabolites included five isolates from the urine and two from the bile. Luteolin is rapidly metabolized, likely in the intestinal lining and/or the liver cells throughout the first-pass metabolism after oral administration (Boersma et al., 2002). Its metabolite, luteolin-3′-O-glucuronide is primarily excreted in the urine. The feces showed no evidence of conjugated metabolites, whereas approximately 5.8% of the starting dose was recovered as free luteolin. A second peak of luteolin and most metabolites occurred between 2 and 8 h after administration, suggesting the possible involvement of enterohepatic recirculation. Biliary excretion is the main route of elimination of orally administered luteolin. In particular, metabolites with 7′-O-glucuronidation show widespread metabolism and extensive distribution of luteolin in rats (Figure 3) (Shi et al., 2024; Wu et al., 2022).

## 3 Pharmacological and therapeutic effects of luteolin

Luteolin has demonstrated a variety of pharmacological influences in both *in vitro* and *in vivo* studies (Huang L. et al., 2023), possessing characteristics of antioxidant (Seelinger et al., 2008a), anti-tumor (Ganai et al., 2021; Seelinger et al., 2008b), anti-microbial (El-Shiekh et al., 2023), anti-viral (Men et al., 2023), anti-inflammatory (Conti et al., 2021; Aziz et al., 2018), anti-apoptotic (Han et al., 2023), anti-allergic (Liang et al., 2020), anti-diabetic (Pradhan and Kulkarni, 2024), chemopreventive (Nguyen et al., 2021), renoprotective (Diniz et al., 2022; Oyagbemi et al., 2021; Xiong et al., 2020), cardioprotective (Liu Z. et al., 2022; Xu et al., 2012) and neuroprotective (Nabavi et al., 2015; Ashaari et al., 2018) properties. Additionally, luteolin has shown anti-anxiety (Gadotti and Zamponi, 2019), antidepressant-like (Mokhtari et al., 2023), antipruritic (Ueda et al., 2005), hepatoprotective (Yao et al., 2023) and antithrombotic (Choi et al., 2015). Numerous studies have indicated that the toxicity of luteolin is minimal, showing no significant harmful effects in clinical trials (Terzo et al., 2023; Gelabert-Rebato et al., 2019; Taliou et al., 2013) (Alzaabi et al., 2022). This included a study by Terzo et al., where 50 obese individuals completed the research protocol. Studies have shown that luteolin’s effect in pre-obesity is safe and has no significant toxic side effects at normal dosages (Terzo et al., 2023). Therefore, the safety of luteolin provides a foundation for further exploration of its use in treating human pulmonary diseases. Some scholars have studied various aspects of luteolin, such as luteolin concentration, lowest toxic dose, and determination of interference effects (Rameshbabu et al., 2024). In terms of Lipinski’s rule of drug-likeness, luteolin meets Lipinski’s Rule of Five, demonstrating drug-like properties (Rameshbabu et al., 2024).

Interestingly, several studies have explored the potential of luteolin to protect lungs across various models. Luteolin also activates the Treg function (Zhang ZT. et al., 2021) and enhances transepithelial sodium transport (Hou et al., 2022) to alleviate inflammation-associated ALI. Here, we discuss the various roles of luteolin in improving dysfunction caused by pulmonary diseases.

### 3.1 Impact of luteolin on ALI/ARDS

**FIGURE 4:**
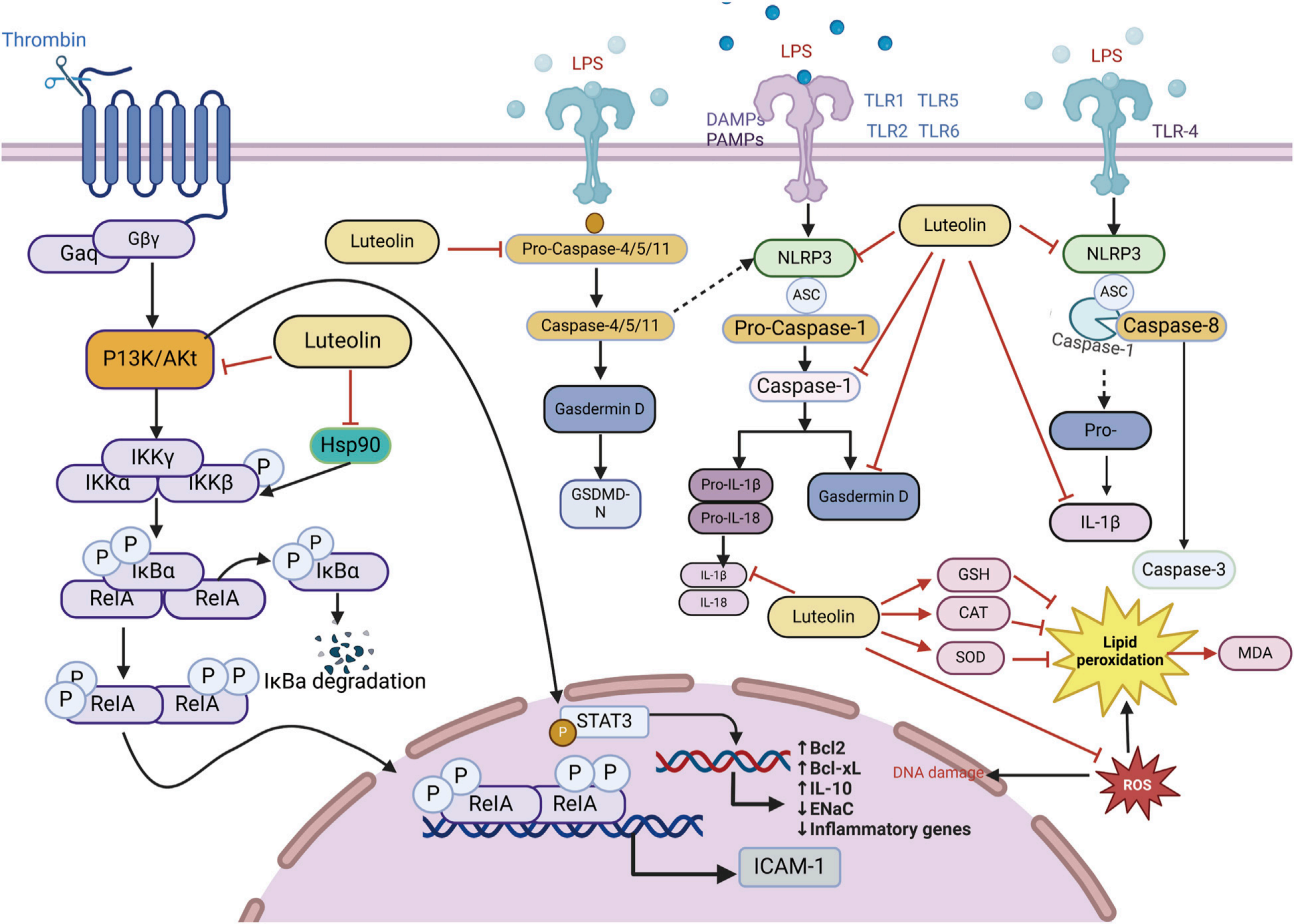
An illustration of the signaling pathways by which luteolin affects ALI/ARDS, created using Biorender.com. The symbol “——|” represents inhibition, and “——>” represents promotion, “----→” represents indirect promotion (PI3K, phosphatidylinositol 3-kinase; GSH, Glutathione, Reduced; STAT3, signal transducer and activator of transcription 3; AKT, Protein kinase B; IL-18, interleukin-18; IL-1β, interleukin-1 beta; IL-10, interleukin-10; TNF-α, tumor necrosis factor; MDA, malondialdehyde; CNP, C-type natriuretic peptide; NLRP3, NOD-like receptor protein 3; SOD, serum superoxide dismutase; CAT, catalase; NF-κB, nuclear factor kappa-B; GSDMD-N, gasdermin D-N).

ALI/ARDS has consistently been a primary cause of acute respiratory exhaustion in seriously ill patients, with high incidence and mortality rates over the past 20 years (Fielding-Singh et al., 2018). According to the “International Sepsis Consensus 5.0 Guidelines” (fifth edition, 2021) (Evans et al., 2021), sepsis is a multi-organ dysfunction caused by a dysregulated host inflammatory response to infection (Cavaillon and Chrétien, 2019; Cecconi et al., 2018). The lungs are the most affected and vulnerable target organs in the process of multiple organ dysfunction caused by sepsis (Fan and Fan, 2018). The pathophysiological process of sepsis-associated acute lung injury (SALI) involves disruption in epithelial and endothelial cell function, excessive accumulation and activation of immune cells, inflammatory response, oxidative stress, programmed cell death, and mobilization of coagulation pathways (Sun et al., 2023). Given the relatively high mortality rate and medical burden, ARDS interventions have always been at the forefront of critical care. Because current treatments for ALI are limited and face additional challenges, the introduction of alternative or complementary treatment methods is necessary (Patel et al., 2018). Fortunately, several “conventional” pharmacological therapies for ALI have been found to reduce mortality. Considering that luteolin is expected to have good effects on ALI, numerous studies have utilized luteolin to explore various cell and animal models induced by Lipopolysaccharide (LPS), demonstrating that luteolin exerts its effects on LPS-induced ALI by acting on multiple pathways involved in the pathogenesis of LPS-induced ALI (Figure 4).

#### 3.1.1 Anti-inflammatory action of luteolin in ALI/ARDS

Studies using animal models of severe sepsis have shown that inflammation plays a key role in the pathophysiology of sepsis. Sepsis is associated with the activation of pro-inflammatory mediators, among which, NF-κB is an important pro-inflammatory transcription factor that mediates the upregulation of several pro-inflammatory cytokines and chemokines, such as tumor necrosis factor (TNF-α), interleukin-6 (IL-6), interleukin-8 (IL-8), and IL-1β, leading to further amplification of inflammatory damage. Luteolin exhibits anti-inflammatory effects by blocking the activity of Hsp90 in macrophages (Malhotra et al., 2001; Yeo et al., 2004; Chen et al., 2014). Thus, luteolin reduces the triggering of LPS-induced MAPK and NF-κB pathways. IL-10, an anti-inflammatory cytokine, can increase sepsis scores and mortality by diminishing inflammatory defenses and dampening immune responses (Levy et al., 2001; Sabat et al., 2010). The anti-inflammatory effects of luteolin are associated with reduced IL-10 levels. Luteolin protects lung tissue from SALI and reduces the excessive accumulation of IL-10 in the lungs during sepsis (Rungsung et al., 2018; Wang X. et al., 2021). Luteolin is a protective antagonist against LPS-induced ALI in mice. Pretreatment of septic mice significantly reduced the mRNA expression of ICAM-1. There is a protective impact on mice with SALI by curbing the ICAM-1, NF-κB, and specific inducible nitric oxide synthase (iNOS) signaling cascades and reducing the levels of pulmonary IL-6, IL-1β, and TNF-α (Rungsung et al., 2018). Luteolin activates Tregs to promote IL-10 expression and alleviates caspase-11-dependent pyroptosis in SALI, thereby significantly inhibiting inflammation and mitigating cecal ligation and puncture (CLP)-induced lung injury (Zhang Z. T. et al., 2021). Luteolin also regulates AKT1/iNOS levels to promote pyroptosis (Zhang et al., 2023). Therefore, luteolin has immense potential for treating ALI, and the pathways above can serve as therapeutic targets for reducing the levels of inflammatory cytokines induced by sepsis (Table 1).

**TABLE 1:** Experimental studies on the anti-inflammatory effects of luteolin on ALI/ARDS.

Dose range	Minimum active concentration	Model	Control	Duration	Extract type	Effect	References
0.5 mg/kg and 1 mg/kg Luteolin	1 mg/kg	*in vivo* BALB/c mice	The negative control: LPS treatment of mice	7days	Purchased from Sigma and dissolved in ethyl alcohol at 10 mM stock solutions	Hsp90↓; c-Jun↓; Akt↓; HMGB1 mediated inflammatory cascade (NF-αB and NO)↓	Chen et al. (2014)
6.25–100 µM Luteolin	50 µM	*in vitro* RAW 264.7 cells	The positive control: HA-Hsp90 plasmids RAW 264.7 cells the negative control: cells untreated or treated only with the vehicle (ethanol)	90 min			
0.2 mg/kg Luteolin		*in vivo* adult Swiss Albino male mice (25–30 g)	The negative control: Group-I, Sham operated; the positive control: Group- III sepsis (CLP)	1 h before CLP surgery	s purchased from Cayman Chemicals, United States of America. Luteolin stock was prepared in absolute alcohol and further diluted with isotonic normal saline	Wet weight/dry weight ratio ↓; ICAM-1 mRNA and NF-kappa B protein expression ↓; Improved lung tissue structure (IL-6 and IL-1β ↓); MDA↓; SOD and CAT↑; PMN infiltration ↓	Rungsung et al. (2018)
20 mg/kg		In Vivo Eight-week-old male C57BL/6 mice	The positive control: CLP group mice; the negative control: Sham group mice	Injected 1 h before CLP.	Purchased from MedChemExpress	(IL-1β, TNF-α)↓; pyroptosis↓; AKT1; iNOS protein↑	Liu and Su (2023)
0–140 μM Luteolin	80 μM	In Vitro RAW264.7 macrophage cells	The negative control: Control group, the cell culture medium contains 0.1% DMSO; the positive control: LPS group, cells stimulated with 1 μg/mL LPS for 6 h	6 h			

#### 3.1.2 Antioxidant role of luteolin in ALI/ARDS

Oxidative stress, characterized by an imbalance between ROS production and the antioxidant defense system, plays a critical role in inflammation-related diseases. Excessive ROS can induce cellular damage, impair normal physiological functions, trigger the release of inflammatory mediators, exacerbate inflammatory responses, and ultimately lead to irreversible tissue damage. Gu et al. coordinated Ce ions with luteolin to synthesize cerium-luteolin nanocomplexes (CeLutNCs). The prepared CeLutNCs can effectively eliminate excess ROS, prevent apoptosis, downregulate the levels of important inflammatory cytokines, modulate the response of inflammatory macrophages, and inhibit the activation of the NF-κB pathway. This confirmed the therapeutic potential of CeLutNCs in an ALI model (Gu et al., 2024). Luteolin alleviates ALI in a mouse model of CLP-induced sepsis through its antioxidant effects (Rungsung et al., 2018; Liu and Su, 2023). Elevated levels of TNF-α can increase free radicals (Cinar et al., 2019; Kutlu et al., 2020), and early studies have shown that luteolin can reduce TNF-α levels in sepsis and other inflammatory experimental models (Rungsung et al., 2018; Basu et al., 2016; Móritz et al., 2024). Celebi and colleagues found that luteolin mainly reduces the expression of TNF-α mRNA, decreases oxidative stress, and reduces the biochemical and histopathological damage occurring in lung tissue of CLP-induced septic rats, which is attributed to the antioxidant properties of luteolin. An increase in malondialdehyde (MDA) caused by free radicals formed during sepsis was normalized by luteolin treatment, which improved glutathione (GSH) levels, CAT, and superoxide dismutase (SOD) activity, and reduced MDA levels. Luteolin is a potent antioxidant even under intense free radical production conditions, such as sepsis (Rungsung et al., 2018; Xiong et al., 2017). Through the mechanisms above, luteolin can affect pulmonary oxidative stress and enhance the activity of antioxidant enzymes, including CAT, glutathione peroxidase, and superoxide dismutase, thereby counteracting lipid peroxidation.

#### 3.1.3 Effect of luteolin on alveolar sodium channels in ALI/ARDS

The main characteristic of ALI/ARDS is pulmonary edema. Rungsung et al. treated mice with luteolin, which not only reduced the wet-to-dry weight ratio and protein concentration in the bronchoalveolar lavage fluid (BALF) but also alleviated lung edema in LPS-challenged ALI mice [77]. ENaC, located on the epithelial cell membrane, is a major participant in sodium transport across the alveoli and a primary determinant of alveolar fluid clearance (AFC). ENaC is crucial in effectively clearing excess alveolar edema fluid, which is vital for restoring gas exchange and reducing damage to peripheral tissues. Luteolin enhances alveolar sodium transport, which is reduced by LPS in H441 cell monolayers, strongly suggesting that luteolin increases sodium transport associated with ENaC activity. Inhibition of cyclic guanosine monophosphate (cGMP) or PI3K can counteract the luteolin-induced increase in ENaC protein expression in the plasma membrane, indicating that the regulation of ENaC by luteolin is related to the cGMP/PI3K pathway. The downstream signaling cascade of PI3K is associated with several effector proteins, including AKT, serum, and glucocorticoid-induced kinase (SGK), both of which can upregulate cellular ENaC by inhibiting the ubiquitination of Nedd4-2 (Hou et al., 2022; Lamothe and Zhang, 2013). Therefore, luteolin alleviates SALI/ARDS by activating alveolar ENaCs via the cGMP/PI3K pathway. Additionally, Chen et al., by knocking down STAT3, demonstrated that luteolin decreases phosphorylation in the JAK/STAT pathway while elevating SOCS3 levels, thereby eliminating the suppressive effect of LPS-induced ENaC expression, indicating that luteolin can alleviate ALI/ARDS by enhancing transepithelial sodium transport through the JAK/STAT pathway (Chen et al., 2023). In summary, luteolin can exert ameliorative effects on LPS-induced ALI through various pathways, such as acting as an anti-inflammatory drug (that inhibits the activity of Hsp90, reduces IL-10 levels, and inhibits NF-κB and MAPK pathways) and an antioxidant (that eliminates excess ROS and enhances antioxidant enzyme activity), as well as improving the function of the pulmonary alveolar ENaC (that activates alveolar ENaC and regulates JAK/STAT pathway) in LPS-induced ALI. Research on luteolin in the treatment of ALI/ARDS provides new insights into the pathophysiology of ALI/ARDS and offers a scientific basis for developing new therapeutic strategies. As our research progresses, we hope to identify luteolin as a novel treatment option for patients with ALI/ARDS.

### 3.2 Impact of luteolin on pneumonia

**FIGURE 5:**
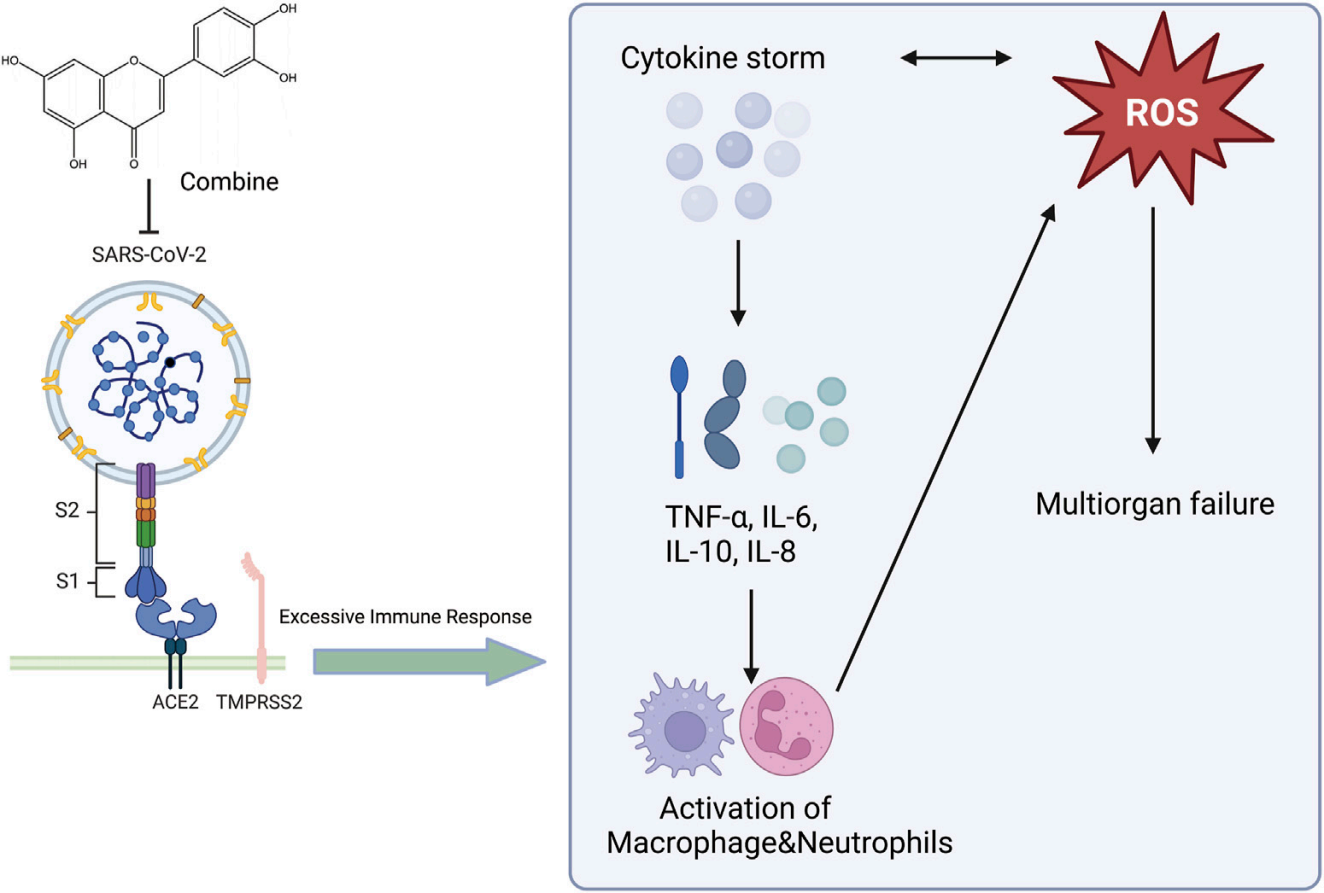
An illustration of the effect of luteolin on the novel coronavirus, created using Biorender.com. The symbol “——|” represents inhibition, and “——>” represents promotion (SARS-CoV-2, Severe Acute Respiratory Syndrome Coronavirus 2; ROS, Reactive Oxygen Species; TNF-α, Tumor Necrosis Factor-alpha; IL-6, Interleukin-6; IL-10, Interleukin-10; IL-8, Interleukin-8; ACE2, Angiotensin-Converting Enzyme 2; TMPRSS2, Transmembrane Protease Serine 2).

Respiratory viruses are a substantial threat to human wellbeing, and respiratory syncytial virus (RSV) is the primary cause of respiratory infections. Studies by Weifeng Li et al. have shown that luteolin exerts therapeutic effects on RSV pneumonia through various mechanisms. Luteolin mitigates lung inflammation in mice, curbs RSV proliferation in the lungs, elevates pulmonary IFN-β and interferon-stimulated genes (ISG) expression, and modulates murine glucose metabolism. AMs constitute the chief producers of IFN-β in the lungs at the beginning of RSV infection, and the absence of AMs can exacerbate RSV-induced lung tissue damage. Luteolin predominantly targets AMs. By safeguarding the mitochondria in AMs and curbing oxidative stress within cells, luteolin can prevent RSV-induced apoptosis and stimulate IFN-β secretion by AMs (Li W. et al., 2024). Luteolin exerts anti-inflammatory effects on lung tissue in rat models of acute pneumonia. Results from the study by Kong et al. indicated that luteolin inhibits inflammation by suppressing cAMP-phosphodiesterase (cAMP-PDE) activity and the expression of microvascular endothelial cell adhesion molecules (Kong et al., 2019). *S. aureus* infections can range from mild skin infections to acute diseases such as pneumonia (David and Daum, 2010). Yuan et al. showed that the expression of genes involved in virulence and biofilm formation in *S. aureus* strains was downregulated after treatment with luteolin, confirming the potential anti-virulence effects of luteolin (Yuan et al., 2022). Additionally, Yuan et al. found that luteolin treatment mitigated the damage to human alveolar epithelial A549 cells caused by the wild-type (WT) strain and protected mice from pneumonia induced by the WT strain. They suggested that luteolin is a promising metabolite that can interfere with the agr system and can be developed as a novel therapeutic agent for *S. aureus* infections (Lv et al., 2023). Moreover, docking studies with methicillin-resistant *Staphylococcus aureus* (MRSA) strains showed that luteolin exhibits good pharmacokinetics, drug-likeness, and high binding energy with MRSA strains. Ligands with good binding energy values, pharmacokinetics, and drug-likeness have been proven to be potential ligands for treating MRSA infections, and luteolin may serve as a potential therapeutic inhibitor of pneumonia caused by MRSA (Nandhini et al., 2023). At the end of 2019, an outbreak of novel acute viral pneumonia caused by severe acute respiratory syndrome coronavirus 2 (SARS-CoV-2) triggered a severe medical and economic crisis. SARS-CoV-2 uses the receptor-binding domain (RBD) of its spike protein to bind to angiotensin-converting enzyme 2 (ACE2) in host cells (Zhu et al., 2023). Therefore, metabolites that bind to human ACE2 or the SARS-CoV-2 spike protein may block the SARS-CoV-2-ACE2 receptor interaction (Day et al., 2021). Luteolin has been shown to bind and significantly inhibit SARS-CoV infection (Stalin et al., 2022). Stalin et al. used computational simulations and virtual screening methods to demonstrate that luteolin engages in uniquely strong interactions with hotspots in the RBD of the SARS-CoV-2 RBD/ACE2 complex, inhibiting the infection of SARS-CoV-2 (Figure 5) (Stalin et al., 2022). In summary, luteolin and its derivatives demonstrate therapeutic potential through multiple mechanisms, such as inhibiting cAMP-PDE activity and the expression of adhesion molecules on microvascular endothelial cells, enhancing pulmonary IFN-β and ISG expression while regulating glucose metabolism in mice, protecting AMs mitochondria and suppressing intracellular oxidative stress to prevent RSV-induced cell apoptosis and stimulate AMs secretion of IFN-β, enhancing the blockade of the RBD/ACE2 complex to inhibit SARS-CoV-2 infection, and downregulating gene expression in *S. aureus* strains. These actions collectively contribute to anti-inflammatory effects, protection of AMs, inhibition of pathogen proliferation, metabolic regulation, and direct interference with virus-host cell receptor interactions, showing promise in treating respiratory diseases. This not only provides a scientific basis for the development of luteolin as a new type of anti-pneumonia drug but also deepens our understanding of the potential of plant-derived metabolites in modern medicine. We look forward to future clinical trials that can further verify these findings and explore new uses for luteolin in treating pneumonia.

### 3.3 Impact of luteolin on asthma

**FIGURE 6:**
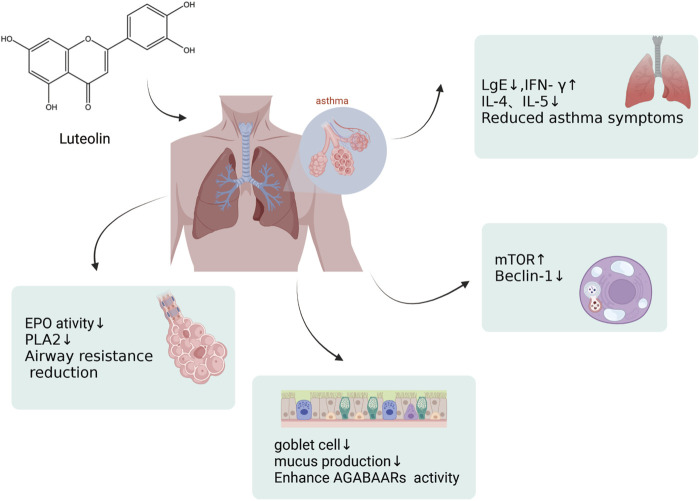
An illustration of effects by which luteolin affects asthma, created using Biorender.com. The symbol “↓” indicates downregulation, and “↑” indicates upregulation (mTOR, mammalian Target of Rapamycin; PLA2, Phospholipase A2; GABAAR, γ-Aminobutyric acid sub-type A receptors).

Asthma is a common chronic airway disease characterized by airway narrowing, thickening of the airway walls, and increased airway mucus secretion, which leads to restricted airflow. The clinical features include dyspnea, wheezing, chest tightness, and cough. The prevalence and incidence of asthma has increased significantly. Asthma affected approximately 262 million people in 2019 and was responsible for 455,000 deaths that year. The asthma mortality rate is highest in low and middle sociodemographic index (SDI) countries, whereas its prevalence is highest in high-SDI countries (GBD 2019 Diseases and Injuries Collaborators, 2020). During the sensitization process and after sensitization in a model of ovalbumin (OVA)-sensitized airway hyperresponsive male BALB/c mice aged 8–9 weeks, the administration of luteolin (0.1 mg/kg body weight led to a marked reduction in OVA-induced constriction of the airways and bronchial hyperreactivity, in contrast to the control group. Luteolin also reduced the levels of OVA-specific IgE in the serum, increased the levels of interferon-gamma (IFN-γ), and decreased the levels of interleukin-4 (IL-4) and interleukin-5 (IL-5) in the BALF, attenuating the asthmatic characteristics of the experimental mice (Das et al., 2003). Luteolin alleviates bronchoconstriction and airway hyperresponsiveness in OVA-sensitized mice. Luteolin exerts anti-asthmatic activity against OVA-induced pulmonary inflammation by downregulating the transcription of CD4^+^ T cells with Th2 phenotype cytokines and inhibiting the production of Prostaglandin E2 (PGE2) (Jin et al., 2009). Luteolin attenuates the immediate and late asthmatic responses to nebulized OVA exposure in conscious guinea pigs. Compared to the control group, luteolin, and apigenin significantly reduced specific airway resistance in both immediate and late asthmatic responses. They also reduced the recruitment of leukocytes and release of histamines, as well as the activity of phospholipase A2 (PLA2) and eosinophil peroxidase (EPO) in bronchoalveolar lavage fluid (BALF). Still, the anti-asthmatic activity of luteolin was lower than that of sodium cromoglycate and dexamethasone (Lee et al., 2014). Therefore, luteolin can be used not only as a lead molecule for identifying effective anti-asthmatic therapies but also as a means of identifying novel anti-asthmatic targets (Figure 6). In addition, luteolin reduces autophagy in allergic asthma. They observed that luteolin treatment significantly inhibited OVA-induced inflammatory responses and autophagy in the lung tissue. Moreover, luteolin activated the PI3K/Akt/mTOR pathway in the lung tissues of asthmatic mice and inhibited the Beclin-1-PI3KC3 protein complex. This strategy encompasses the prevention of autophagic processes in allergic asthma via upregulation of the PI3K/Akt/mTOR pathway and downregulation of the Beclin-1-PI3KC3 complex (Wang S. et al., 2021). Asthma often manifests as an excessive buildup of mucus in the airway lining, which can lead to severe clinical outcomes. Although general asthma therapies are effective, specialized treatments designed to limit mucus overproduction in patients with asthma are still lacking. Recent studies have revealed that stimulating the gamma-aminobutyric acid type A receptor (GABAAR) is crucial for promoting excessive mucus secretion by pulmonary airway epithelial cells. Shen and colleagues found that in an OVA-induced asthma mouse model, with a 10 mg/kg administration, luteolin markedly reduced the number of goblet cells within pulmonary tissue and suppressed excessive mucus production. Luteolin hindered GABAAR-induced currents in A549 cells, according to patch-clamp data. Additionally, the suppressive effects of luteolin on goblet cell hyperplasia and excessive mucus secretion induced by OVA are reversed by picrotoxin, a GABAAR antagonist (Shen et al., 2016). In summary, luteolin and its derivatives have demonstrated potential in treating asthma by modulating various cellular signaling pathways and immune responses. These include the activation of the PI3K/Akt/mTOR pathway and inhibition of the Beclin-1-PI3KC3 protein complex. The therapeutic potential is reflected in mechanisms such as reducing airway inflammation, decreasing airway hyperresponsiveness, modulating immune cytokines, reducing eosinophil infiltration, inhibiting autophagy, and suppressing excessive mucus secretion. After evaluating the potential impact of luteolin on asthma, we believe that this metabolite shows promise for improving asthma symptoms by modulating various cellular signaling pathways and immune responses. Nonetheless, we should recognize the complexity of asthma, which necessitates further research to determine the optimal dose of luteolin for asthma treatment. We are confident that with a deeper understanding of the mechanisms of action of luteolin, it could become an important metabolite in future asthma treatment strategies.

### 3.4 Impact of luteolin on COPD

**FIGURE 7:**
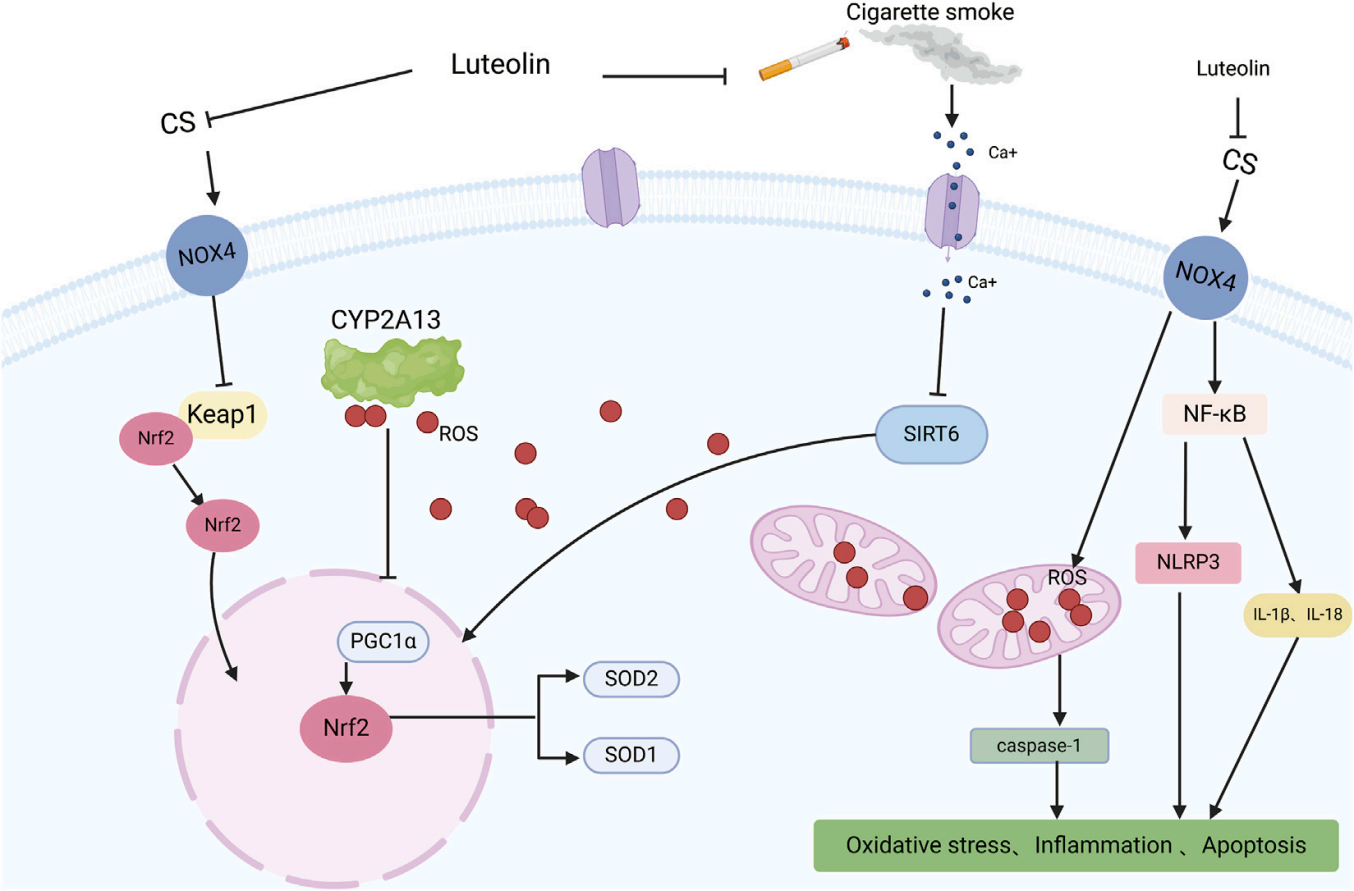
An illustration of the protective effect of luteolin in CS-induced COPD, created using Biorender.com. The symbol “——|” represents inhibition, and “——>” represents promotion (CS, Cigarette Smoke; NOX4, NADPH oxidase 4; Ca+, Calcium ions; CYP2A13, Cytochrome P450 family 2 subfamily A member 13; Keap1, Kelch-like ECH-associated protein 1; NF-KB, Nuclear factor kappa B; ROS, Reactive Oxygen Species; SIRT6, Sirtuin 6; Nrf2, Nuclear factor erythroid 2-related factor 2; NLRP3, NLR family pyrin domain containing 3; IL-1β, Interleukin-1 beta; IL-18, Interleukin-18; PGC1α, Peroxisome proliferator-activated receptor gamma coactivator 1-alpha; SOD2, Superoxide dismutase 2; SOD1, Superoxide dismutase 1).

COPD is one of the leading causes of morbidity and mortality worldwide (Buhl et al., 2024) and poses a significant economic and social burden (Alupo et al., 2024). Patients experience progressive deterioration until end-stage COPD, which is characterized by severe airflow limitations, severely compromised lung function, chronic respiratory failure, advanced age, multiple comorbidities, and severe systemic manifestations/complications. COPD is often underdiagnosed and undertreated (Viegi et al., 2007). Intensified oxidative stress and body-wide inflammation are critical elements of the complex pathophysiology of COPD (Austin et al., 2016). Studies have shown that matrix metalloproteinases (MMPs) are associated with COPD progression, and luteolin has been identified as an inhibitor of MMP-2 and MMP-9 (Ende and Gebhardt, 2004). Therefore, Yaghi et al. explored the use of luteolin in their study, measuring ciliary beat frequency (CBF) in nasal cilia from COPD patients as an important indicator of ciliary function in COPD. Compared to the high-risk and control groups, CBF was significantly reduced in individuals with moderate-to-severe COPD. The treatment group observed a significant and sustained increase in CBF in COPD cilia tested with luteolin, increasing the CBF of cilia in patients with COPD, favorably enhancing mucociliary clearance, reducing retention of secretions, and decreasing infections (Yaghi et al., 2012). Li et al. proposed that luteolin inhibits inflammation and oxidative stress in COPD by suppressing the NADPH oxidase 4 (NOX4)-mediated NF-κB signaling pathway. Studies have used cigarette smoke (CS) to induce COPD in mice or A549 cells both *in vivo* and *in vitro*. *In vivo*, luteolin mitigated the inhibitory effects of CS on body weight and the activities of SOD and CAT in COPD model mice and reduced the levels of MDA. Similar results have been obtained *in vitro*. Li et al. transfected A549 cells with a NOX4 overexpression plasmid to induce NOX4 overexpression. Compared to the control group, the overexpression of NOX4 reversed the downregulation of the ratio of p-p65/p65 and p-IκB/IκB in the luteolin group, upregulation of oxidative stress-related proteins, and reduction of inflammatory-related factors. Luteolin alleviates inflammation, oxidative stress, and activation of the NOX4-mediated NF-κB signaling pathway induced by CS in A549 cells (Li M. et al., 2023). Zhou and colleagues proposed that luteolin alleviates oxidative stress induced by CS in COPD by modulating the transient receptor potential vanilloid type 1 (TRPV1) and cytochrome P450 family 2 subfamily A member 13 (CYP2A13)/nuclear factor erythroid 2-related factor 2 (NRF2) signaling pathways. They observed that luteolin downregulated the expression of TRPV1 and CYP2A13 proteins and upregulated the expression of sirtuin 6 (SIRT6) and NRF2 proteins in CS + LPS-induced COPD mice and A549 cells treated with CS. CS treatment increased intracellular calcium ions, overall ROS, and mitochondria-derived ROS levels in A549 cells. Importantly, luteolin inhibited the influx of Ca^2+^ into CS-treated A549 cells and mitigated the excessive production of mitochondrial and intracellular ROS. By modulating the TRPV1/SIRT6 and CYP2A13/NRF2 signaling pathways, luteolin exhibits a protective effect by reducing oxidative stress and inflammation in COPD mice induced by CS-and LPS, as well as in A549 cells exposed to CS (Figure 7) (Zhou et al., 2023). In summary, luteolin has the potential to alleviate inflammation and oxidative stress and improve cilia function in COPD by inhibiting NOX4-mediated NF-κB signaling pathway, regulating TRPV1 and CYP2A13/NRF2 signaling pathways, and modulating various signaling pathways and cytokines. However, we are also aware that COPD is a multifactorial disease requiring a comprehensive treatment strategy. Therefore, future research should explore the combined application of luteolin and other drugs to manage COPD effectively.

### 3.5 Impact of luteolin on pulmonary fibrosis

Idiopathic pulmonary fibrosis (IPF), classified as progressive fibrosing interstitial lung disease (ILD), leads to a gradual decline in lung function (Raghu et al., 2018). The hallmarks of IPF include a progressive deterioration in lung capacity, exacerbation of fibrosis as depicted on high-resolution computed tomography (HRCT) scans, decline in both symptomatology and quality of life for patients, and a propensity for early death (Travis et al., 2013; Wells et al., 2018; Cottin et al., 2019; Kolb and Vašáková, 2019). Aside from pirfenidone and nintedanib, which are specifically used for treating IPF, no other medications have been approved for treating ILDs (Brown et al., 2020). The predominant pharmacological approach for treating other progressive fibrosing ILDs involves immunosuppression (Cottin et al., 2019). When the fibrotic response of an ILD to lung injury advances to a stage where fibrosis becomes self-perpetuating and continues to progress, targeted anti-fibrotic therapy is essential to mitigate the rate of disease progression. Clinical studies have demonstrated that natural substances extracted from plants possess potent antifibrotic properties and can alleviate dyspnea, a common and debilitating symptom experienced by patients with ILD (Chang et al., 2021). Luteolin is an active flavonoid metabolite isolated from *Lonicera japonica* and is known for its broad range of biological activities, particularly its antioxidant and anti-inflammatory effects. Ren and colleagues loaded luteolin into γ-cyclodextrin metal-organic frameworks (CD-MOFs) for pulmonary drug delivery using a dry powder inhaler. *In vitro* lung deposition results showed that luteolin effectively inhibited the progression of ILD in bleomycin (BLM)-induced fibrotic ILD rat model. Compared to the control group, treatment with luteolin significantly protected rats from BLM-induced lung injury. They demonstrated that luteolin possesses potent anti-inflammatory activity and that inhalation of luteolin could be considered a promising new strategy for treating fibrotic ILD (Ren et al., 2023). Oral administration of luteolin (10 mg/kg) in both the early and delayed luteolin treatment groups effectively inhibited the infiltration of neutrophils into BALF and increased TNF-R and IL-6 levels in C57BL/6 mice intubated with BLM. Luteolin also mitigated collagen deposition, TGF-β1 expression, and pulmonary fibrosis following BLM injection. Subsequently, *in vitro* studies showed that luteolin inhibited the expression of TGF-β1-induced reactive serum myoglobin (R-SMA), type I collagen, and vimentin in primary cultured mouse lung fibroblasts. Luteolin significantly blocked the downregulation of epithelial markers (E-cadherin) and upregulation of mesenchymal cell markers (fibronectin and vimentin) mediated by TGF-β1 and maintained the epithelial morphology of human alveolar epithelial-derived A549 cells. This indicates that luteolin exerts effective antifibrotic activity by inhibiting pulmonary inflammation and suppressing myofibroblast differentiation and the epithelial-mesenchymal transition (EMT) of cells (Chen et al., 2010). Data analysis in informatics implied that luteolin may offer substantial therapeutic benefits in treating IPF and gastroesophageal reflux disease (GERD). By identifying and analyzing differentially expressed genes (DEGs) between IPF and GERD and predicting drug molecules for IPF and GERD, luteolin was recognized as a potential biomarker and promising therapeutic target for both IPF and GERD (Table 2) (Shen et al., 2016). In summary, luteolin demonstrates potential in the treatment of IPF through various mechanisms, including anti-inflammatory effects, anti-fibrotic actions (inhibition of Smad3 phosphorylation in the TGF-β1 signaling pathway), inhibition of myofibroblast differentiation, suppression of EMT, modulation of key signaling pathways, and potential as a drug delivery vehicle. After analyzing the potential applications of luteolin in treating pulmonary fibrosis, we were encouraged by the antifibrotic potential of the metabolite. We believe that luteolin’s anti-inflammatory and anti-fibrotic properties and its role in modulating key signaling pathways make it a strong candidate for treating pulmonary fibrosis. Future studies should focus on determining the optimal route of administration and luteolin dosage to maximize its therapeutic effects.

**TABLE 2:** Experimental studies of luteolin in the treatment of pulmonary fibrosis.

Model	Does	Duration	Type of study	Effect	References
Male C57BL/6 rats	10 mg/kg Luteolin	21 day	in vivo	Collage deposition↓; TGF-β1 mRNA↓; myocardium inflammation (TNF-α, IL-6, NUET) ↓	Chen et al. (2010)
Primary lung fibroblasts and A549 cells	25 μM Luteolin	72 h	in vitro	TGF-β1 expression (R-SMA, Collagen 1 and vimentin) ↓; P-Smad 3↓	
BLM-induced fibrosing ILD rats	400 mg/kg LUT + CDMOF	20 days	in vivo	weight↑; lung coddicient↑; TGF-β1 and Smad3↓	Brown et al. (2020)
MHS cells	0.5,1 and 2 ug/mL LUT + CDMOF	12 h	in vitro	myocardium inflammation (TNF-α, IL-1β and IL-6) ↓	

### 3.6 Therapeutic effect of luteolin on lung cancer

The GLOBOCAN 2020 estimates from the International Agency for Research on Cancer (IARC) regarding cancer incidence and mortality rates show that carcinoma of the lung stands as the most prevalent cause of death from cancer, with projections indicating approximately 2.2 million new instances and 1.8 million deaths worldwide (Sung et al., 2021). In China, the primary treatment options for lung cancer include surgical intervention, radiation treatment, chemotherapy, precision cancer therapies, immune system treatments, and traditional Chinese medicine (Yang et al., 2018; Chaft et al., 2016). Recent reports suggest that luteolin may be a novel and potent agent against lung cancer with the potential to inhibit disease progression (Imran et al., 2019).

KRAS mutations are known to increase the expression of programmed death-ligand 1 (PD-L1) within tumor cells, enabling the tumor to shun the immune system for detection and subsequent destruction. An *in vitro* experiment demonstrated that luteolin inhibits the growth of KRAS-mutated cells in a dose-dependent manner. The expression of PD-L1 is a consequence of STAT3 activity, which engages the PD-L1 gene promoter upon phosphorylation. Luteolin inhibits the STAT3 signaling pathway and the expression of PD-L1, thereby exposing tumor cells to T cells of the immune system (Jiang et al., 2021; Watterson and Coelho, 2023). MiR-34a is commonly downregulated in cancer cells, and elevated levels of miR-34a have been shown to suppress tumor growth (Zhang et al., 2019; Fu et al., 2023). Research has indicated that luteolin upregulates the expression of miR-34a, which may subsequently inhibit the proliferation of non-small cell lung cancer (NSCLC) cells (Shi et al., 2014; Jiang et al., 2018). Circular RNAs (circRNAs) are upregulated in lung cancer tissues, and luteolin suppresses lung cancer progression by targeting the circ_0000190/miR-130a-3p/notch-1 signaling pathway (Zheng et al., 2023). Luteolin may inhibit the proliferation and migration of A549 cells by reducing the expression of AR and modulating the phosphorylation of AR receptor sites (Li X. et al., 2023).

The anticancer properties of luteolin have also been demonstrated through the induction of apoptosis in neoplastic cells (Çetinkaya and Baran, 2023). Luteolin induces mitochondria-dependent apoptosis in human lung adenocarcinoma cells (Chen et al., 2012). Luteolin promotes the activation of caspase-9 and caspase-3, decreases the expression of Bcl-2, increases the expression of BAX, increases the BAX/Bcl-2 ratio, and induces apoptosis by activating intrinsic pathways (Chen et al., 2012). Additionally, the accumulation of ROS induced by luteolin can enliven the extrinsic pathways modeled by TNF-induced signaling (Ju et al., 2007). Luteolin has been demonstrated to inhibit EMT and downregulate E-cadherin by suppressing the triggering of key signaling pathways, such as TGF-β1 and the PI3K/AKT-NF-κB-Snail pathways (Chen et al., 2013). Furthermore, Masraksa and colleagues have demonstrated that luteolin can hinder the spread and invasion of lung cancer cells by targeting the signaling pathways of focal adhesion kinase and non-receptor tyrosine kinase (Src/FAK) as well as its downstream pathways involving Ras-related C3 botulinum toxin substrate 1 (Rac1), cell division cycle 42 (Cdc42), and ras homologous gene family member A (RhoA) (Masraksa et al., 2020). Luteolin works synergistically with osimertinib, a third-generation epidermal growth factor receptor tyrosine kinase inhibitor (EGFR-TKI), to overcome acquired resistance in NSCLC cells by suppressing the hepatocyte growth factor (HGF)-MET-Akt signaling cascade, which is involved in MET amplification and hyperactivation (Huang G. et al., 2023). When used alone or in combination with factor-related apoptosis-inducing ligand (TRAIL), a novel and potent anticancer agent, can enhance the expression of death receptor 5 (DR5) and promote dynamin-related protein 1 (Drp1)-dependent mitochondrial fission. By increasing DR5 expression and Drp1-mediated mitochondrial fission, luteolin enhances the sensitivity of NSCLC cells to TRAIL (Wu et al., 2020). Another study indicated that LIM Kinase 1 (LIMK1) is a target of luteolin, which suppresses tumor growth by inhibiting LIMK1 activity (Zhang M. et al., 2021). Beyond its anticancer characteristics, luteolin also possesses the ability to alleviate bone cancer pain (Zhou et al., 2022). Luteolin involves numerous determinants and cascades related to lung cancer progression (Rakoczy et al., 2023). In summary, luteolin has shown potential as a therapeutic agent for lung cancer by affecting multiple key factors in disease progression, and further research into its clinical applications is warranted (Table 3). Luteolin has shown the potential to inhibit lung cancer progression by affecting various key tumor biological pathways. We believe that these mechanisms of action provide a scientific basis for luteolin as a candidate drug for lung cancer treatment. Future research should focus on clinical trials of luteolin and optimizing its efficacy through drug delivery systems.

**TABLE 3:** Experimental studies of luteolin in the treatment of lung cancer cells.

Model	Does	Duration	Effect	References
Human NSCLC A549 and H460 cells	0,10,20 and 40 μM Luteolin	24 h	Tumor suppressor P53 and P21 expression↑; apoptosis (Bax, caspase-3, caspase-9) ↑; cancer cells proliferation↓	Jiang et al. (2018)
H358, H460 and H549 cells	30 μM Luteolin	24 h	MUC1/STAT3 signaling↓; block PD-1/PD-L1 axis IL-2↑	Jiang et al. (2021)
A549 cells	80 μM Luteolin	48 h	AR protein expression↓; cell proliferation↓; lung cancer cell apoptosis↑; migration ofA549 is inhibit	Li et al. (2023b)
A459, H1975 cells and Beas-2B cells	20 μM Luteolin and/or 25 ng/mL TRAIL	24 h	DR5 expression↑; Drp1↓; apoptosis↑; p-JNK↑	Wu et al. (2020)
NCI-H1975 and NCI-H1650 cells	5,10,20 and 40 μM luteolin	24,48 or 72 h	LIMK1 signaling pathway↓; p-LIMK↓; P-cofilin↓; CyclinD1 and D3↓	Zhang et al. (2021b)

## 4 Limitations and future perspectives

The multifaceted pharmacological profile of luteolin makes it a potential candidate for further investigation as a natural therapeutic agent for the treatment of pulmonary diseases. However, poor solubility in water and low bioavailability hinder their therapeutic potential. This article emphasizes that strategies to enhance water solubility and bioavailability through chemical modifications and advanced pharmaceutical formulations such as nanovesicles and cyclodextrin complexes hold promise for addressing the challenges associated with luteolin. Balancing the concentration of luteolin, determining the minimum toxic dose, and addressing interference effects in broad-spectrum assays are also issues that deserve attention. While preclinical studies in cellular and animal models are promising, and numerous studies indicate that luteolin has minimal toxicity, this article emphasizes the need for further research, particularly clinical trials in humans and rigorous toxicity experiments that are still required to assess its safety and promote its rational development, to fully understand the efficacy and safety of luteolin in treating pulmonary diseases. Owing to the structural diversity of flavonoids in plants and the inherent limitations of existing technologies, industrial-scale production of flavonoids faces challenges that necessitate the improvement and optimization of current extraction techniques (Li L. et al., 2024). In this context, enhancing the purification efficiency and recovery rate of luteolin is a critical step that is essential for the large-scale production and widespread application of luteolin-based pharmaceuticals. Although there are already commercial luteolin health foods and cosmetic products on the market (Wang Z. et al., 2021), the use of luteolin as a drug for the treatment of other diseases is still in the clinical trial phase (Shi et al., 2024; Di Stadio et al., 2022; Cordaro et al., 2020). Future research should focus on two key areas: optimizing the solvent extraction technology for luteolin and developing advanced pharmaceutical formulations that can improve its solubility and bioavailability. These two tasks are of great significance in overcoming the limitations of luteolin in practical applications, fully exerting its therapeutic effects, and promoting its development for clinical applications.

## 5 Conclusion

In this article, we reviewed the therapeutic potential and molecular mechanisms of action of luteolin in various pulmonary diseases. Luteolin has shown significant effects in the treatment of ALI/ARDS, COPD, asthma, pulmonary fibrosis, and lung cancer, owing to its pharmacological actions such as anti-cancer, antioxidant, and anti-inflammatory properties. Despite its notable bioactivity, water-solubility and bioavailability of luteolin limit its clinical applications. These limitations can be overcome through chemical structural modifications and pharmaceutical formulation innovations. This article summarizes the research progress in improving the solubility and bioavailability of luteolin, discusses its multifaceted therapeutic effects in the treatment of pulmonary diseases and provides new directions for future research.

## Glossary

PGE2: Prostaglandin E2
NF-κB: Nuclear Factor kappa-B
COPD: Chronic Obstructive Pulmonary Disease
ALI: Acute Lung Injury
ARDS: Acute Respiratory Distress Syndrome
ENaC: Epithelial Sodium Channel
AFC: Alveolar Fluid Clearance
cGMP: Cyclic Guanosine Monophosphate
LPS: Lipopolysaccharide
IL-6: Interleukin-6
TNF-α: Tumor Necrosis Factor alpha
CLP: Cecal Ligation and Puncture
ROS: Reactive Oxygen Species
GSH: Glutathione
SOD: Superoxide Dismutase
MDA: Malondialdehyde
HMGB1: High-Mobility Group Box 1
iNOS: Inducible Nitric Oxide Synthase
GSDMD-N: Gasdermin D-N gasdermin D
TRAIL: Tumor Necrosis Factor-related Apoptosis-inducing Ligand
DR5: Death Receptor five
NLRP3: NOD-like Receptor Protein 3
LIMK1: LIM Kinase 1
NSCLC: Non-Small Cell Lung Cancer
EGFR: Epidermal Growth Factor Receptor
PD-L1: Programmed Death-Ligand 1
STAT3: Signal Transducer and Activator of Transcription 3
miR-34a: MicroRNA-34a
circRNA: Circular RNA
Tregs: regulatory T cells
IPF: Idiopathic pulmonary fibrosis
ILD: interstitial lung disease
PLA2: Phospholipase A2
GABAAR: γ-Aminobutyric acid sub-type A receptors
CS: Cigarette Smoke
NOX4: NADPH oxidase four
Ca+: Calcium ions
CYP2A13: Cytochrome P450 family 2 subfamily A member 13
Keap1: Kelch-like ECH-associated protein 1
SIRT6: Sirtuin 6
Nrf2: Nuclear factor erythroid 2-related factor 2
PGC1α: Peroxisome proliferator-activated receptor gamma coactivator 1-alpha
SOD2: Superoxide dismutase 2
SOD1: Superoxide dismutase 1
ACE2: angiotensin-converting enzyme 2
TMPRSS2: Transmembrane Protease serine 2
ISG: interferon-stimulated genes
MRSA: methicillin-resistant *staphylococcus aureus*
SARS-CoV-2: severe acute respiratory syndrome coronavirus.
